# Minority ethnicity patient satisfaction and experience: results of the National Cancer Patient Experience Survey in England

**DOI:** 10.1136/bmjopen-2016-011938

**Published:** 2016-06-28

**Authors:** Richard J Pinder, Jamie Ferguson, Henrik Møller

**Affiliations:** 1 Department of Primary Care and Public Health, School of Public Health, Imperial College London, London, UK; 2 Faculty of Life Sciences and Medicine, Division of Health and Social Care Research, King's College London, Guy's Hospital, London, UK; 3 Cancer Epidemiology, Population and Global Health, King's College London, Guy's Hospital, London, UK

**Keywords:** patient experience, patient satisfaction, quality, cancer, health services

## Abstract

**Objectives:**

This study sought to explore the differential patient satisfaction reported by patients with cancer who are from ethnic minority backgrounds, examining patient-reported experience of interacting with medical and nursing staff.

**Setting:**

As a secondary analysis, we collated data collected over two consecutive annual rounds of the National Cancer Patient Experience Survey (NCPES) from September 2012 to November 2013.

**Participants:**

There were 138 878 responses from 155 hospital trusts across the National Health Service in England, representing a response rate of 63.9% based on the total identified cohort of patients receiving cancer care over those 2 years.

**Outcomes:**

We used the results of the annual survey, which sought to assess overall patient satisfaction along with patient experience of interacting with clinical nurse specialists, hospital doctors and ward nurses.

**Results:**

Ethnic minority patients reported lower satisfaction and less positive experiences of care overall. While some of this difference appeared related to demographic and socioeconomic variation, ethnic minority patients remained less positive than those in the White British group, after statistical adjustment. Ethnic minority patients also reported lower confidence in, and less understanding of, healthcare professionals, including clinical nurse specialists, doctors and ward nurses.

**Conclusions:**

Given the diversity of the British population, as well as the clustering of ethnic minority patients in certain urban areas, a better understanding of the expectations and additional needs of ethnic minority patients is required to improve their experience of and satisfaction with cancer care.

**Strengths and limitations of this study**

- Using data from two consecutive years of the National Cancer Patient Experience Survey, this study examines the lower satisfaction and poorer experience reported by ethnic minority patients, who each year represent a small, but increasing, proportion of the British population.
- Non-response remains an issue, and it is not possible to determine to what extent non-response bias may affect these results.
- Self-reported ethnicity is a proxy for several factors including linguistic capability as well as differing cultural norms and expectations.

## Introduction

Over the past 20 years, feedback from patients has increasingly been recognised as a core metric of healthcare quality.^1^ ^2^ Considerable research has been undertaken to interpret how patients may form their conclusions about the quality of care they receive.^3–7^ Patient satisfaction typically describes a patient's overall feeling towards care, whereas patient experience seeks to minimise the effect of expectation and to avoid value judgements.^8^ Notwithstanding the absence of a commonly agreed definition of satisfaction,^9^ how this term relates to experience and how these concepts correlate with other measures of health system quality remain topics of debate.^9^ ^10^

In the UK, health service bodies (including the National Health Service, NHS) commission a range of patient surveys spanning primary and secondary care. On the basis of recommendations made about cancer care in 2007,^11^ and with the system-wide focus on patient experience in 2008,^2^ the first National Cancer Patient Experience Survey (NCPES) was undertaken in 2010. Since 2012, the survey has become an important annual review of the quality of cancer care provided across all NHS hospitals in England.

On the basis that patient-reported satisfaction and experience are affected by the patient's own characteristics as well as those of the care provider,^12^ it is important for us to consider both aspects when interpreting results from patient surveys. A number of patient characteristics have been observed to affect satisfaction and experience: women tend to report less positively,^13^ as do younger adults.^14^ ^15^ Patients from poorer socioeconomic backgrounds tend towards more negative reviews,^16–18^ as do those with poorer health status.^13^ While patient-side factors may influence survey responses to some degree, providers and health system planners must not lose sight of the potential for provider-side quality issues to influence reporting. Moreover, in a truly patient-centred system, there remains an important principle to improve satisfaction and experience, and to respond to patient need no matter how diverse.

The proportion of the UK population reporting their ethnicity as White British or White Irish has decreased from 91.3% to 86.0% from 2001 to 2011 (the most recent UK census).^19^ Often focused in metropolitan areas, the non-White British groups form the majority in a number of areas: in boroughs (local administrative districts) of East London, the White British population is 16.7%.^19^ In the USA, an analysis of experience of patients with cancer found non-White ethnicity as a primary predictor of lower patient satisfaction.^20^ Previous published work on the English 2011/2012 Cancer Patient Experience Survey (CPES) data has been consistent with these findings.^21^ The concept of ethnicity is not straightforward: ethnicity by self-report may overlap with individuals' ideas of self-identity, spanning nationality, religion and language. Ethnicity is recorded differently in each country, but may be useful within a country in facilitating an equitable and patient-centred health service. Qualitative research into experience of patients with cancer in Australia has shown that immigrant patients face additional challenges for their diagnosis, including cultural isolation and linguistic hurdles.^22^ Recently, research has suggested that immigrant patients (in particular those from Chinese backgrounds) report poorer experience than those identifying as Anglo-Australian;^23^ in that study both linguistic and cultural expectations were suggested to be involved.

In studying ethnicity through this national survey, the relatively small number of ethnic minority patients in the survey is a challenge for statistical power. By bringing together two consecutive annual surveys, we aim to identify associations of ethnicity on patient satisfaction with, and experience of, cancer care in the English health service.

## Methods

### Data collection and collation

We combined the data sets from the 2013 and 2014 surveys (table 1). For the variables used in this study, there was no difference between the 2013 and 2014 questionnaires. The samples included all patients receiving treatment for cancer over a 3-month period in the preceding year. The surveys included both, inpatients and day-case/outpatients, corresponding to approximately one-third and two-thirds of the sample size, respectively. These data sets were anonymised at source and, therefore, as a secondary analysis of national data, explicit ethical approval for the study was not required.

**Table 1:** Survey summaries for the National Cancer Patient Experience Surveys (NCPES) 2013 and 2014

	NCPES 2013^24^	NCPES 2014^25^ ^26^ ^34^
Variable (%)		
Patients treated during sample period	116 525	118 081
Actual sample size*	*Not reported*	109 763
Respondents	68 737 (63.9%)	70 141 (63.9%)
Hospital Trusts†	155	153
Response rate range per Trust	33–74%	42–75%
Sample inclusion period	September 2012 to November 2012	September 2013 to November 2013
Proportion of respondents rating their care as excellent or very good	58 525 (88.4%)	59 677 (89.0%)

*The actual sample size excludes patients known to have died, patients opting out for a variety of reasons and to those to whom no questionnaire has been sent.

†Hospital Trusts are the organisations providing care, where a Trust may be responsible for providing care across two or more hospital sites; mergers account for the reduction in Trust numbers between 2013 and 2014.

For the 2013 survey, 68 737 questionnaires were returned corresponding to a response rate of 63.9%.^24^ A 63.9% response rate was also achieved for 2014, totalling 70 141 responses.^25^ The response rates exhibited considerable variation between hospitals, ranging from 33% to 75%. Reasons for non-response were not provided, but there was a tendency for lower response rates among London hospitals.^24^ This response rate was broadly similar to other NHS surveys.^26^

### Demographic measures

Sex, employment status and ethnicity were self-reported through the paper questionnaire. The Index of Multiple Deprivation (IMD), the official composite measure of deprivation in England, was derived on the basis of patient postcode ascertained from the health record; we refer to this as ‘deprivation’ in this paper. Whether a patient was treated in London or not was identified for each record on the basis of hospital provider location.

### Patient satisfaction measure

The overall patient satisfaction question was the final question to be asked after up to 69 other questions, and enquired, ‘Overall, how would you rate your care?’ Respondents subsequently ticked one of five responses, labelled ‘excellent’, ‘very good’, ‘good’, ‘fair’ or ‘poor’. For most of the analyses (and unless otherwise specified), we grouped the responses as ‘excellent’ and ‘not excellent’. An additional sensitivity analysis combining ‘excellent’ and ‘very good’ versus the remaining three responses was also undertaken.

### Patient experience measures

Five other questions regarding patient experience were selected to determine associations between ethnicity and other factors. These five questions were chosen *a priori* on the basis of their being related to hospital-based care, and involving the respondents' interactions and perceptions of three discrete professional groups; we are not aware of any previous literature specifically using these questions. We hypothesised that patient interaction with these three professional groups may impact on satisfaction and would potentially be of value in identifying training needs, with a view to improving the quality of healthcare provided.

The first of these groups comprised the clinical nurse specialists (CNS), a group of nursing staff who provide continuous specialist support to cancer patients. Participants were asked, ‘When you have important questions to ask your CNS, how often do you get answers you can understand?’ Respondents subsequently ticked one of four responses, labelled ‘all or most of the time’, ‘some of the time’, ‘rarely or never’ or ‘I do not ask any questions’. Similar questions were asked regarding hospital doctors and ward nurses. For the purposes of the analysis, we recoded the responses into either ‘all or most of the time’ and ‘not all or most of the time’.

For both, hospital doctors and ward nurses, a second question asked was, ‘Did you have confidence and trust in [staff group] treating you?’ Respondents were invited to tick a box corresponding to one of three responses: ‘in all of them’, ‘in some of them’ or ‘in none of them’. For the purposes of the analysis, we recoded the responses into either ‘in all of them’ or ‘not in all of them’.

### Data analyses

The data were compiled and analysed using STATA V.14.0. Descriptive analyses were supplemented by univariate and multivariate logistic regression calculating odds ratios (ORs) and 95% confidence intervals (CIs). Where applicable to the logistic regression, p values for trend or heterogeneity were calculated. Analyses were conducted on the basis of the responses recorded: for example, if a patient did not interact with a ward nurse during his or her care pathway, they would be omitted from the analysis (and not included in the denominator) for that analysis.

## Results

### Principal findings

A total of 138 878 responses were collated into a single data set. Substantial differences were noted between the ethnicities (table 2). The group identifying as White British accounted for 86.8% of the sample size, of which 10.1% were treated in London hospitals. By comparison, the other non-White ethnicities were generally younger, and substantially more likely to live in a deprived neighbourhood (where higher IMD indicates higher deprivation). A large proportion of these non-White groups lived in London.

**Table 2:** Baseline characteristics of ethnic groupings, by sex, age, socioeconomic, employment status and place of treatment

	n (%)	Male (%)	Median age	(IQR)	Median IMD	(IQR)	Retired (%)	Treated in London (%)
Participants	138 878 (100%)	46.9	68	(59–75)	14.2	(8.5–24.3)	63.0	13.4
White								
British	115 875 (86.8)	47.2	68	(60–76)	13.9	(8.4–23.4)	65.0	10.1
Irish	1094 (0.8)	48.7	70	(63–76)	18.5	(10.0–31.6)	70.2	38.5
Any other White background	3277 (2.5)	42.6	64	(53–73)	18.0	(10.4–29.9)	48.8	44.1
Black								
African	574 (0.4)	39.7	57	(48–67)	32.5	(20.0–44.2)	30.1	69.3
Caribbean	908 (0.7)	47.0	68	(56–76)	31.2	(20.9–42.7)	58.4	60.1
White and Black African	76 (0.1)	40.8	61	(50–68)	27.0	(14.4–38.9)	38.8	44.7
White and Black Caribbean	123 (0.1)	40.7	62	(52–72)	27.3	(15.7–43.1)	45.6	26.8
Any other Black background	313 (0.2)	44.7	57	(49–70)	31.6	(20.2–40.5)	34.6	69.7
Asian								
Bangladeshi	121 (0.1)	56.3	54	(45–64)	38.2	(22.2–51.8)	25.5	64.5
Indian	1102 (0.8)	43.4	63	(53–72)	20.4	(12.4–31.8)	45.2	44.9
Pakistan	424 (0.3)	47.2	59	(46–68)	30.1	(18.0–45.2)	30.9	24.5
White and Asian	106 (0.1)	35.9	57	(48–67)	17.8	(8.2–28.6)	31.4	33.0
Any other Asian background	603 (0.5)	39.0	59	(48–68)	20.6	(12.7–32.6)	34.3	61.0
Other								
Chinese	281 (0.2)	40.6	58	(48–66)	20.5	(10.4–34.8)	35.6	43.1
Any other mixed background	166 (0.1)	36.1	56	(47–67)	20.5	(12.5–29.5)	31.0	38.0
Any other ethnic group	1088 (0.8)	40.1	62	(52–70)	19.4	(10.4–33.7)	42.6	47.2
Unknown	7322 (5.5)	45.7	67	(58–73)	12.7	(7.7–22.1)	58.0	14.0

The IMD (2010) is the official statistic for neighbourhood-level deprivation in England and ranks each of the 32 482 neighbourhoods in order of deprivation. The IMD is a composite index comprising income, employment, health and disability, education skills and training, barriers to housing, crime and environmental metrics. It does not include ethnicity. It is based on the patient's registered address. In this analysis, the value provided for IMD is the median centile where a higher number indicates a higher level of deprivation.

IMD, Index of Multiple Deprivation.

### Gender, age and socioeconomic status

Women were less likely than men to rate their care as excellent (table 3); this persisted after adjustment for sex, age group, ethnicity, IMD quintile and employment status (adjusted OR 0.95 (95% CI 0.93 to 0.97)). The highest ratings were reported among the largest age group, those 60–74 years of age. Those 90 years and older reported lower rating of overall care compared with the 60–74 years age group (adjusted OR 0.67 (0.61 to 0.77)). Neither before nor after adjustment was deprivation associated with rating of care. The experience of care was poorer among those not employed or those who had retired (adjusted OR 0.91 (0.87 to 0.95)).

**Table 3:** Sociodemographic characteristics of population and overall satisfaction with NHS cancer care, with univariate and multivariate logistic regression, n=133 265

	Excellent				
	n	OR	(95% CI)	AOR*	(95% CI)
Sex (%)					
Male	35 797 (57.4)	1.00		1.00	
Female	39 376 (55.5)	0.92	(0.91 to 0.95)	0.95	(0.93 to 0.97)
		p Value	<0.001	p Value	<0.001
Age group (years)					
Under 30	521 (53.4)	0.83	(0.73 to 0.95)	0.88	(0.77 to 1.01)
30–44	2920 (52.7)	0.81	(0.77 to 0.86)	0.88	(0.82 to 0.93)
45–59	14 745 (54.2)	0.85	(0.83 to 0.88)	0.88	(0.85 to 0.92)
60–74	36 068 (57.9)	1.00		1.00	
75–89	19 306 (56.5)	0.94	(0.92 to 0.97)	0.94	(0.92 to 0.97)
90+	669 (48.9)	0.69	(0.62 to 0.77)	0.67	(0.61 to 0.77)
		p Value†	<0.001	p Value†	0.80
Ethnic group					
*White*					
British	63 806 (57.4)	1.00		1.00	
Irish	617 (59.0)	1.07	(0.94 to 1.21)	1.06	(0.93 to 1.20)
Any other White background	1516 (48.4)	0.70	(0.65 to 0.75)	0.71	(0.66 to 0.76)
*Black*					
African	203 (37.7)	0.45	(0.38 to 0.54)	0.48	(0.40 to 0.58)
Caribbean	318 (37.3)	0.44	(0.38 to 0.51)	0.45	(0.39 to 0.52)
White and Black African	32 (42.7)	0.55	(0.35 to 0.87)	0.69	(0.42 to 1.12)
White and Black Caribbean	60 (50.9)	0.77	(0.53 to 1.10)	0.78	(0.54 to 1.14)
Any other Black background	122 (40.7)	0.51	(0.40 to 0.64)	0.52	(0.41 to 0.65)
*Asian*					
Bangladeshi	33 (28.2)	0.29	(0.20 to 0.44)	0.33	(0.22 to 0.50)
Indian	381 (36.1)	0.42	(0.37 to 0.48)	0.43	(0.37 to 0.49)
Pakistan	145 (34.9)	0.40	(0.33 to 0.49)	0.42	(0.34 to 0.52)
White and Asian	43 (41.4)	0.52	(0.35 to 0.77)	0.51	(0.34 to 0.77)
Any other Asian background	233 (40.2)	0.50	(0.42 to 0.59)	0.53	(0.44 to 0.63)
*Other*					
Chinese	97 (36.3)	0.42	(0.33 to 0.54)	0.45	(0.35 to 0.58)
Any other mixed background	80 (51.0)	0.77	(0.56 to 1.06)	0.80	(0.58 to 1.10)
Any other ethnic group	492 (47.2)	0.66	(0.59 to 0.75)	0.69	(0.60 to 0.78)
		p Value‡	<0.001	p Value‡	<0.001
IMD quintile					
First (least deprived)	18 323 (57.0)	1.03	(0.99 to 1.06)	1.01	(0.98 to 1.05)
Second	18 006 (56.9)	1.02	(0.99 to 1.05)	1.00	(0.97 to 1.04)
Third	15 946 (56.4)	1.00		1.00	
Fourth	12 622 (55.6)	0.97	(0.93 to 1.00)	1.00	(0.96 to 1.03)
Fifth (most deprived)	9817 (55.4)	0.96	(0.92 to 1.00)	1.03	(0.99 to 1.07)
		p Value†	<0.001	p Value†	0.77
Employment status					
Full time	12 312 (56.6)	0.97	(0.95 to 1.01)	1.06	(1.02 to 1.11)
Part time	6666 (56.8)	0.99	(0.95 to 1.02)	1.06	(1.01 to 1.11)
Retired	46 793 (57.2)	1.00		1.00	
Other	7730 (52.0)	0.81	(0.78 to 0.84)	0.91	(0.87 to 0.95)
		p Value‡	<0.001	p Value‡	<0.001

*Adjusted for sex, age group, ethnicity, IMD quintile and employment status.

†For trend.

‡For heterogeneity.

AOR, adjusted OR; IMD, Index of Multiple Deprivation; NHS, National Health Service.

### Ethnicity

The proportion of the White British group describing their care as excellent was 57.4%. The most positive patient experience score was reported by respondents identifying themselves as White Irish, although this difference was not statistically significant. Almost all the non-White ethnicities reported poorer experience. While some of the differences were attenuated following adjustment for age, sex, deprivation and employment, a statistically significant difference persisted for several groups. Among Black African and Black Caribbean groups, excellent care was reported by 37.7% and 37.3%, respectively. For both groups, the odds of reporting excellent care were less than half of those reported for the white British group, a difference that persisted after adjustment (adjusted OR 0.48 (0.40 to 0.58) and 0.45 (0.39 to 0.52), respectively).

Low ratings were also reported for other ethnicities: Indian (adjusted OR 0.43 (0.37 to 0.49)); Pakistani (0.42 (0.34 to 0.52)) and Chinese (0.45 (0.35 to 0.58)). The Bangladeshi group was associated with the lowest proportion reporting their care as excellent (adjusted OR 0.33 (0.22 to 0.50)).

The overall rating of care appeared higher among those identifying as being of mixed ethnicity than those from non-mixed minority ethnic backgrounds, but still lower than those from the White British group. However, the mixed ethnic groups were comparatively small in number and, following adjustment, no statistical significance persisted except among those of White/Asian mixed ethnicity (adjusted OR 0.51 (0.34 to 0.77)).

The results of the sensitivity analysis that grouped patients responding to the satisfaction question as ‘excellent’ or ‘very good’ together are reported in the online supplementary material. The results were broadly similar, with several of the results even more extreme among the ethnic minority groupings. The minor differences were that sex was not found to be significantly different after adjustment, while the trend for deprivation was found be statistically significant (after adjustment).

10.1136/bmjopen-2016-011938.supp1 Supplementary material

### Perceptions of staff

Turning to communication with specific healthcare professionals (table 4), it was notable that the White ethnic group (comprising White British, White Irish and White other) reported better experience than the Black and Asian groups (aggregated as in table 3).

**Table 4:** Patient experience metrics by ethnic grouping (see optional presentation of unadjusted ORs in table 1)

	Yes				
	n	OR	(95% CI)	AOR*	(95% CI)
Rated care overall as excellent (%) (Q70)					
White	65 939 (57.1)	1.00		1.00	
Black	735 (39.0)	0.48	(0.44 to 0.53)	0.50	(0.45 to 0.55)
Asian	835 (36.8)	0.44	(0.40 to 0.48)	0.45	(0.41 to 0.50)
Other	669 (45.6)	0.63	(0.57 to 0.70)	0.65	(0.59 to 0.73)
*Total responses: 121 045*		p Value‡	<0.001	p Value‡	<0.001
Always understood CNS (%) (Q24)					
White	79 732 (82.4)	1.00		1.00	
Black	1256 (77.0)	0.72	(0.64 to 0.80)	0.78	(0.69 to 0.88)
Asian	1452 (78.4)	0.77	(0.69 to 0.86)	0.78	(0.69 to 0.88)
Other	1026 (81.6)	0.95	(0.82 to 1.09)	0.97	(0.84 to 1.13)
*Total responses: 101 503*		p Value‡	<0.001	p Value‡	<0.001
Always understood hospital doctor (%) (Q37)					
White	60 724 (76.9)	1.00		1.00	
Black	869 (71.1)	0.74	(0.65 to 0.84)	0.87	(0.76 to 0.99)
Asian	1098 (69.2)	0.68	(0.61 to 0.75)	0.71	(0.64 to 0.80)
Other	766 (72.1)	0.76	(0.68 to 0.89)	0.81	(0.70 to 0.93)
*Total responses: 82 845*		p Value‡	<0.001	p Value‡	<0.001
Always had confidence in and trusted hospital doctor (%) (Q38)					
White	67 288 (84.8)	1.00		1.00	
Black	978 (78.4)	0.65	(0.57 to 0.74)	0.74	(0.64 to 0.86)
Asian	1245 (78.6)	0.65	(0.58 to 0.74)	0.76	(0.67 to 0.87)
Other	856 (80.4)	0.73	(0.63 to 0.85)	0.81	(0.69 to 0.95)
*Total responses: 83 212*		p Value‡	<0.001	p Value‡	<0.001
Always understood ward nurse (%) (Q41)					
White	52 757 (66.9)	1.00		1.00	
Black	721 (58.5)	0.70	(0.62 to 0.78)	0.75	(0.66 to 0.84)
Asian	929 (59.0)	0.71	(0.64 to 0.79)	0.73	(0.66 to 0.81)
Other	676 (63.4)	0.86	(0.76 to 0.97)	0.86	(0.76 to 0.98)
*Total responses: 82 712*		p Value‡	<0.001	p Value‡	<0.001
Always had confidence in and trusted ward nurse (%) (Q42)					
White	55 425 (70.1)	1.00		1.00	
Black	717 (57.8)	0.58	(0.52 to 0.63)	0.58	(0.52 to 0.66)
Asian	950 (60.0)	0.63	(0.58 to 0.71)	0.68	(0.61 to 0.76)
Other	692 (64.8)	0.78	(0.69 to 0.89)	0.82	(0.72 to 0.93)
*Total responses: 82 909*		p Value‡	<0.001	p Value‡	<0.001

Ethnic groups were aggregated into the categories displayed in table 2.

*Adjusted for sex, age group, ethnic group, IMD quintile and employment status.

‡For heterogeneity.

AOR, adjusted OR; CNS, clinical nurse specialist; IMD, Index of Multiple Deprivation.

Black and Asian ethnic groups reported poorer experience across all five of the healthcare professional questions both before and after adjustment (table 1). The White ethnic group reported best understanding among their interactions with the CNS (82.4%) followed by hospital doctors (76.9%) and then ward nursing staff (66.9%). The Black and Asian ethnic groups reported lower understanding in the range of 13–29% in relative terms, across all three professional groups, but with substantially lower understanding for ward nursing staff in absolute terms (58.5% and 59.0%, respectively).

Trust among the Black and Asian ethnic groups was considerably greater for doctors than for ward nursing staff. Among the Black ethnic group, 78.4% of respondents stated they had confidence and trust in all of the hospital doctors compared with 57.8% in ward nursing staff. Similarly, for respondents in the Asian ethnic group, the difference was 78.6% in hospital doctors compared with 60.0% in ward nurses. A smaller difference was observed for the White ethnic group, of 84.8% and 70.1%, respectively.

## Discussion

### Statement of principal findings

Patients from ethnic minority backgrounds receiving cancer care in England report statistically significant poorer satisfaction of overall care as well as poorer experience communicating with specific groups of healthcare professionals. While some of this difference can be attributed to sociodemographic factors, an apparent gap remains in satisfaction and experience of patients with cancer for these ethnic minority patients.

### Ethnicity and epidemiology

That ethnic minority patients report poorer experience with their care may be described by one or more of three general explanations: their reporting is biased or confounded by non-response or other factors; they may have different expectations of care; or they may actually receive objectively poorer care. Work from Australia has already identified some of the challenges that immigrant populations face,^22^ ^23^ and it is notable that those identifying as being of Chinese ethnicity report such low levels of satisfaction in our analysis, albeit with low numbers reporting.

In respect of possible confounding by health status, our study did not examine variation in diagnosis or disease course. There is evidence that patients from ethnic minority backgrounds may present later in breast cancer.^27^ ^28^ Certain cancer types may also be associated with a range of genetic and environmental exposures,^29^ which may confound the association between ethnicity and experience. Yet differences may be influenced by differing expectations of care: it is possible that by adapting care to suit the needs of the majority (White) population, a reverse effect will occur for ethnic minorities.

Similar to the highest ratings being reported among the largest ethnic group, the highest ratings were also reported among the largest age group. Likewise, this may indicate best adaptation of services to the largest ‘customer base’. Yet notable among the findings was the apparently steep drop-off in experience for patients 90 years or older. It is not possible to determine whether a patient completed the questionnaire personally or whether it was done through a proxy. It is possible that for older patients a higher proportion of questionnaires were completed by family members or carers, and this may contribute to the poorer experience reported.

### Ethnicity and communication

A notable success to emerge from the data set is the positive patient experience associated with understanding the CNS. While a drop-off is noted for the minority ethnic groups compared with the White ethnic group, the proportion of ethnic minority patients who understood their CNS all or most of the time was above 75%: this was higher than for hospital doctors or ward nurses. This would suggest that successful communication with minority ethnicity patients is possible, and that perhaps with appropriate continuity of care, training and effort, a better quality of communication is possible for other staff groups.

It is concerning that the rating for ward nursing staff is lower overall than for the other two professional groups. A partial explanation may be that patients are admitted and come into contact with ward nursing staff during the more acute phases of their illness. It is therefore possible that patients are more acutely unwell at these times, and this may account for poorer experience overall. However, several alternative explanations are also possible that may contribute to the differential rating. Nursing care for inpatients seldom occurs on specialist cancer wards, and therefore general ward nurses are less likely to have specialist cancer knowledge. With the advent of CNS it is also possible that CNS recruitment has drawn away cancer expertise from ward nursing, leaving behind a culture that is comparatively de-skilled as nursing specialisms emerge. Ward nursing staff also present challenges for identification, as patients may not differentiate between qualified nurses and healthcare assistants (who are usually less trained ward-based care staff). Accordingly, the assessment of ward nurses may encompass a number of non-nurse clinicians. Finally, nursing staff may also be rated on a number of non-clinical factors including the quality of other services such as provision of food. Yet, none of these explanations clearly account for the poorer reported experience between ethnic groups.

### Strengths and weaknesses

The key strength of this study is that we have been able to quantify, with good statistical power, the associations between ethnicity and a range of patient experience metrics.

It is not possible for us to ascertain the reasons for non-response. First, while a 69.4% response rate is consistent with other surveys of this type, there is a likelihood that non-response bias may mask or exacerbate some effects: less satisfied patients may be less inclined to respond. In terms of statistical precision, estimates provided by the company that runs the survey (Quality Health Ltd) state that the 95% CI is ±0.3% for point estimates for each of the 2 years included.^26^ By compiling 2 years of data, this precision is further improved. Second, the questionnaire involves sections to be ‘skipped’ if the questions are not relevant to the participant. For example, patients seen only as outpatients may not be able to answer questions about ward nurses. In this way, it is not possible to account for question-specific non-response. Question-level response counts are included in table 4.

However, it is at the conceptual level of ethnicity that this study posed its greatest limitation. Ethnicity in this study was collected by a process of self-identification, which is valid. Yet, ethnicity itself can be fluid and may be confounded by issues of language and culture. While one may argue that ethnicity is a proxy for these other factors, to what extent language may present a key barrier to successful communication is uncertain. This is relevant in the context of receiving, managing and judging care, but also in completing the questionnaire. As proposed for older patients who rate care more poorly, it may be family members or other carers who are more likely to complete the questionnaire for patients unable to read English. Likewise, it is possible that language barriers will contribute to non-response. Whether response may be associated with more polarised experience is impossible to determine from the data sets employed.

Previous reports and studies have shown that ethnicity is associated with reported experience although not always negatively.^30^ Previous work in the UK on patient experience and general practice has suggested that ethnic minority patients have a tendency to report poorer experience, although some of this effect was attributed to the clustering of Asian patients in poorly performing urban practices.^31^ However, the same study noted inconsistent findings for the Black ethnic group.

Comparisons between the UK and USA should be treated with caution, due to the material differences in health system design, but also because of the differences in ethnic categorisation (the Asian group in the UK is applied to people of southern Asian extraction, not the Far East). There has also been discussion over whether ethnic minority clustering may contribute to the lower patient experience ratings observed for patients receiving cancer care in London hospitals.^32^

### Implications

The fact that 89% of the White ethnic group rate their care as excellent or very good is a success and should not be ignored. However, ethnic minority populations tend to be clustered (largely in urban areas), meaning that while representing <15% of the population overall, they account for much larger proportions of the patient population in some hospitals. Despite the introduction of advanced communication skills training for senior cancer clinicians within the health service, a better understanding of what ethnic minority patients with cancer expect from cancer care, along with support, is required so that services can be developed to better meet these needs.

The mixed ethnicity group is potentially the most heterogeneous of all, and challenges many of the more traditional attempts at ethnic categorisation. That the mixed groups report experience between that of the White and minority groups may suggest that these groups integrate and absorb the values and attitudes of the majority. The mixed ethnicity group is the fastest expanding ethnicity in the UK. As the proportion of people of mixed ethnicity continues to increase and age in the UK, this group should not be forgotten.^33^

## Conclusion

While limited by the potential for non-response bias, this study suggests that the needs of patients with cancer who are from ethnic minority backgrounds are not being met, and that these needs are under-addressed by existing systems. Better understanding of these unmet needs is required if we are to address this inequality. While ethnic minorities account for approximately an eighth of all patients with cancer, continuing immigration to the UK and an increasingly old existing ethnic minority population will mean that these patients will exert increasing demands on cancer care over the coming years.
