# Supplementary material for: Minority ethnicity patient satisfaction and experience: results of the National Cancer Patient Experience Survey in England
Source: BMJ Open. 2016 Jun 28;6(6):e011938. doi: 10.1136/bmjopen-2016-011938 (PMC4932347; doi:10.1136/bmjopen-2016-011938)
Supplement: Supplementary material [file bmjopen-2016-011938supp_material.pdf]

SUPPLEMENTARY MATERIAL

Minority ethnicity patient satisfaction and experience: the results of the National Cancer Patient Experience Survey in England

Running title: Ethnicity and cancer patient experience in England

10 May 2016

Richard J Pinder[1,2] richard.pinder@doctors.org.uk, +44 7930 314747 -- *corresponding author*
Jamie Ferguson[2] jamie.ferguson@kcl.ac.uk
Henrik Møller[3] henrik.moller@kcl.ac.uk

1. Department of Primary Care and Public Health, School of Public Health, Imperial College London, Reynolds Building, St Dunstans Road, London W6 8RP, United Kingdom.

2. Division of Health and Social Care Research, Faculty of Life Sciences and Medicine, King's College London, 5th Floor, Addison House, Guy's Hospital, London SE1 1UL, United Kingdom.

3. Cancer Epidemiology, Population and Global Health, King's College London, 3rd Floor, Bermondsey Wing, Guy's Hospital, Great Maze Pond, London SE1 9RT, United Kingdom.

Key words: patient experience; quality; cancer; health services.

Supplementary Table 1: Sensitivity analysis; sociodemographic characteristics of population and overall satisfaction with NHS cancer care with univariate and multivariate logistic regression, n = 133,265.

	Excellent or Very Good					
	n		OR	(95% CI)	AOR*	(95% CI)
**Sex (%)**						
- Male	55 528	(89.1)	1.00		1.00	
- Female	62 674	(88.4)	0.93	(0.90-9.96)	0.98	(0.94-1.01)
			p-value	<0.001	p-value	0.29
**Age Group**						
- Under 30	852	(87.4)	0.79	(0.65-0.96)	0.96	(0.78-1.17)
- 30-44	4739	(85.6)	0.68	(0.63-0.74)	0.83	(0.76-0.91)
- 45-59	23 624	(86.8)	0.75	(0.72-0.79)	0.85	(0.81-0.91)
- 60-74	55 875	(89.7)	1.00		1.00	
- 75-89	30 461	(89.2)	0.95	(0.91-0.99)	0.92	(0.87-0.96)
- 90 plus	1160	(84.7)	0.63	(0.55-0.74)	0.62	(0.53-0.72)
			p-value†	<0.001	p-value†	0.64
**Ethnic group**						
White						
- British	99 379	(89.3)	1.00		1.00	
- Irish	934	(89.3)	1.00	(0.82-1.21)	0.99	(0.81-1.21)
- Any other white background	2630	(84.0)	0.63	(0.57-0.69)	0.66	(0.60-0.73)
Black						
- African	419	(77.9)	0.42	(0.34-0.52)	0.51	(0.41-0.64)
- Caribbean	659	(77.3)	0.41	(0.35-0.48)	0.46	(0.39-0.54)
- White and Black African	63	(84.0)	0.63	(0.34-1.16)	0.95	(0.45-1.99)
- White and Black Caribbean	97	(82.2)	0.55	(0.34-0.88)	0.61	(0.38-1.00)
- Any other Black background	234	(78.0)	0.42	(0.32-0.56)	0.47	(0.36-0.62)
Asian						
- Bangladeshi	74	(63.3)	0.21	(0.14-0.30)	0.27	(0.18-0.40)
- Indian	760	(72.0)	0.31	(0.27-0.35)	0.33	(0.28-0.37)
- Pakistan	306	(73.7)	0.33	(0.27-0.42)	0.40	(0.32-0.50)
- White and Asian	85	(81.7)	0.53	(0.32-0.88)	0.60	(0.35-1.01)
- Any other Asian background	444	(76.6)	0.39	(0.32-0.47)	0.46	(0.38-0.57)
Other						
- Chinese	194	(72.7)	0.32	(0.24-0.42)	0.35	(0.26-0.46)
- Any other mixed background	130	(82.8)	0.57	(0.38-0.87)	0.65	(0.42-1.00)
- Any other ethnic group	852	(81.7)	0.53	(0.45-0.62)	0.61	(0.51-0.71)
			p-value‡	<0.001	p-value‡	<0.001
**IMD Quintile**						
- First (least deprived)	28 901	(90.0)	1.14	(1.08-1.20)	1.11	(1.05-1.17)

- Second	28 268	(89.3)	1.06	(1.00-1.12)	1.04	(0.98-1.09)
- Third	25 099	(88.7)	1.00		1.00	
- Fourth	19 889	(87.5)	0.89	(0.84-0.94)	0.94	(0.89-0.99)
- Fifth (most deprived)	15 358	(86.6)	0.82	(0.78-0.87)	0.93	(0.88-0.99)
			p-value†	<0.001	p-value†	<0.001
**Employment status**						
- Full time	19 242	(88.4)	0.87	(0.85-0.93)	0.98	(0.92-1.05)
- Part time	10 459	(89.1)	0.95	(0.89-1.01)	1.00	(0.93-1.09)
- Retired	73 325	(89.6)	1.00		1.00	
- Other	12 544	(84.4)	0.63	(0.60-0.66)	0.74	(0.70-0.79)
			p-value‡	<0.001	p-value‡	<0.001

* Adjusted for sex, age group, ethnicity, IMD quintile and employment status.
† - For trend; ‡ - For heterogeneity
